# Common Considerations for Genotoxicity Assessment of Nanomaterials

**DOI:** 10.3389/ftox.2022.859122

**Published:** 2022-05-24

**Authors:** Rosalie K. Elespuru, Shareen H. Doak, Andrew R. Collins, Maria Dusinska, Stefan Pfuhler, Mugimane Manjanatha, Renato Cardoso, Connie L. Chen

**Affiliations:** ^1^ Division of Biology, Chemistry and Materials Science, Office of Science and Engineering Laboratories, Center for Devices and Radiological Health, U.S. Food and Drug Administration, Silver Spring, MD, United States; ^2^ Institute of Life Science, Swansea University Medical School, Swansea, United Kingdom; ^3^ Department of Nutrition, Institute of Basic Medical Sciences, University of Oslo, Blindern, Norway; ^4^ Health Effects Laboratory, Department of Environmental Chemistry, NILU-Norwegian Institute for Air Research, Kjeller, Norway; ^5^ Global Product Stewardship, Human Safety, Procter & Gamble Mason Business Centre, Mason, OH, United States; ^6^ Division of Genetic and Molecular Toxicology, Food and Drug Administration, National Center for Toxicological Research, Jefferson, AR, United States; ^7^ Millipore Sigma, Rockville, MD, United States; ^8^ Health and Environmental Sciences Institute, Washington, DC, MD, United States

**Keywords:** nanomaterials, genotoxicity, methods, mutagenicity, clastogenicity, biocompatibility

## Abstract

Genotoxicity testing is performed to determine potential hazard of a chemical or agent for direct or indirect DNA interaction. Testing may be a surrogate for assessment of heritable genetic risk or carcinogenic risk. Testing of nanomaterials (NM) for hazard identification is generally understood to require a departure from normal testing procedures found in international standards and guidelines. A critique of the genotoxicity literature in Elespuru et al., 2018, reinforced evidence of problems with genotoxicity assessment of nanomaterials (NM) noted by many previously. A follow-up to the critique of problems (what is wrong) is a series of methods papers in this journal designed to provide practical information on what is appropriate (right) in the performance of genotoxicity assays altered for NM assessment. In this “Common Considerations” paper, general considerations are addressed, including NM characterization, sample preparation, dosing choice, exposure assessment (uptake) and data analysis that are applicable to any NM genotoxicity assessment. Recommended methods for *specific assays* are presented in a series of additional papers in this special issue of the journal devoted to toxicology methods for assessment of nanomaterials: the *In vitro* Micronucleus Assay, TK Mutagenicity assays, and the *In vivo* Comet Assay. In this context, NM are considered generally as insoluble particles or test articles in the nanometer size range that present difficulties in assessment using techniques described in standards such as OECD guidelines.

## Introduction

Engineered nanomaterials (NM) can have biological effects that differ from those of materials with the same chemical composition, as a result of size, shape, and surface area or surface chemistry. Such differences may include altered biological activity such as uptake, distribution or biological interactions. The small size leads to increased surface area relative to the mass of the particle, which could affect biological disposition and interactions. These same physical and chemical properties may impact the genotoxicity assays designed to assess the potential hazard of NM (Dusinska et al., 2017; ISO, 2017; Elespuru et al., 2018; Faria et al., 2018).

The methods considerations provided in this and accompanying papers are a follow-up to those addressed earlier by Elespuru et al. (2018), in critiques of the issues and problems in the published literature on genotoxicity assessment of NM. The lack of reliable publications related to accurate hazard identification and risk assessment of NM causes problems, especially related to cancer risk assessment.

As noted by others and summarized in Elespuru et al. (2018), some of the problems relate to aspects of the tests that need to be adjusted for assessment of NM, due to interference of nano-sized materials with the test or the endpoint, or lack of uptake of particles into the target cells. Other issues relate to the lack of standard systems, e.g., specific cell lines, used generally for genotoxicity studies, as opposed to myriad cell systems found in the literature that may yield uninterpretable results. Thus, the Common Considerations document and accompanying methods for specific assays are models for genotoxicity testing and assessment of NMs. As noted by others and summarized in Elespuru et al. (2018), bacterial (Ames) reverse mutation assays are not recommended for assessment of NM; thus, a protocol for this assay is not included. This Common Considerations paper consists of a set of issues to be addressed relative to methods and approaches common to the genotoxicity assays, including material characterization, sample preparation, metabolic activation (if needed), dose selection, exposure assessment (e.g., uptake) and data assessment.

**FIGURE 1:**
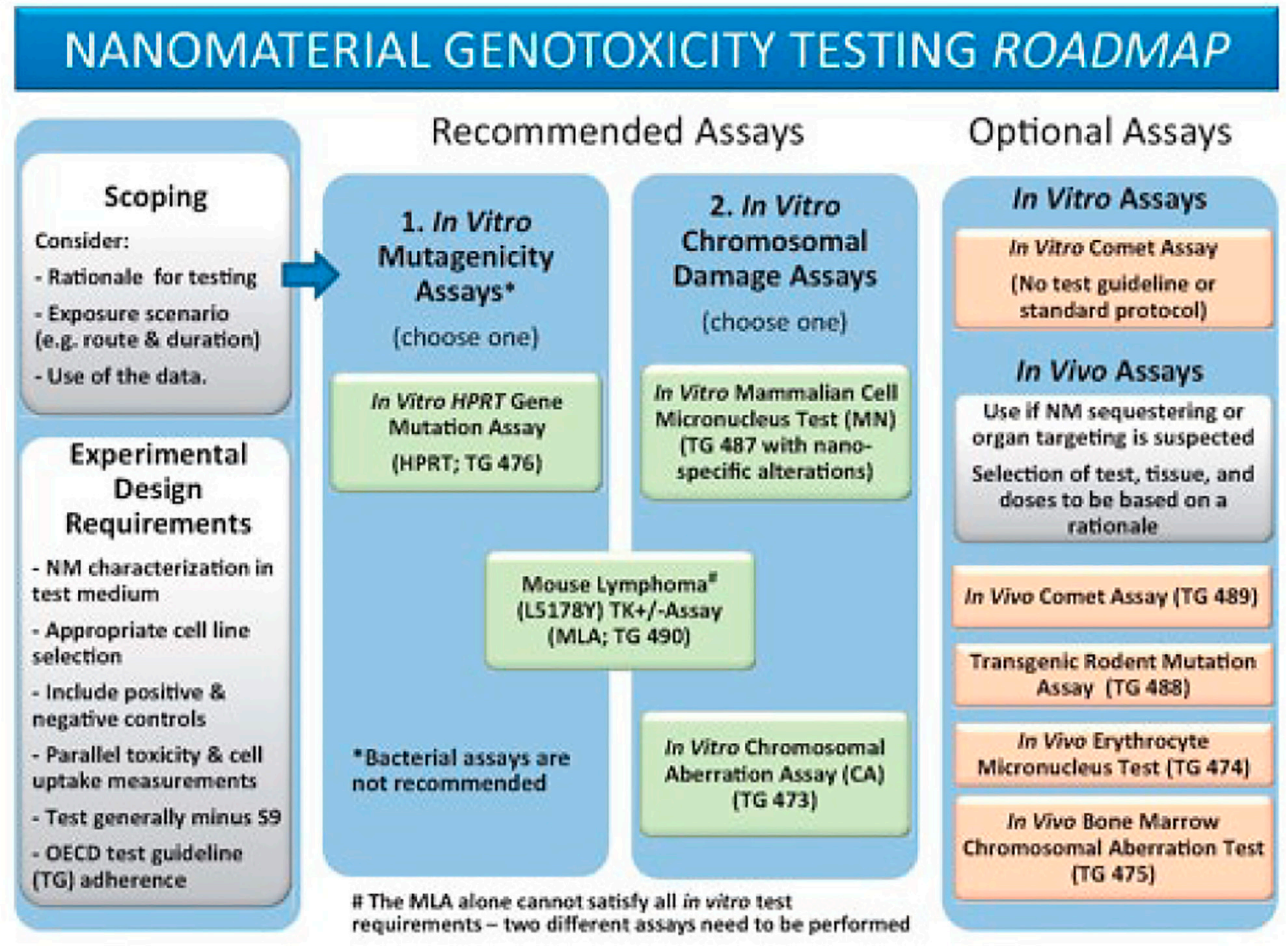
Reproduced from Elespuru et al. (2018), p. 393, with permission from Oxford University Press.

The following parameters are considered common considerations for any of the genotoxicity tests recommended in the Toxicological Sciences Roadmap (Figure 1) and should accompany the methods on the *In Vitro* Micronucleus Assay, the thymidine kinase (TK)-based mutagenicity assays, and the *In Vivo* Comet Assay.

## Methods Considerations

### Integrating Information From Other Tests, Including Animal Assays

Toxicity testing *in vivo* is invaluable for obtaining information on biodistribution, accumulation, and clearance of NM that cannot be assessed using *in vitro* assays. If data from these studies are available, attention should be paid to the features of these *in vivo* effects, especially regarding tissue or organ sequestering of NM (part of “scoping”, Figure 1) (Elespuru et al., 2018). Agglomeration or aggregation characteristics of NM in the *in vitro* and *in vivo* tests are also important to consider as they may interfere with the assay or cause unexpected effects. NM are generally not soluble in aqueous media and may be present as suspensions during the test.

### Nanomaterial Description

The source of the NM should be provided, i.e., purchased (source) or manufactured/synthesized at the researcher’s institution. A physical description of the NM would include chemical composition, structure (size and shape), surface chemistry (where relevant), and an assessment of material or particle diversity, preferably accompanied by a microscopic image. Other features that could be described, if known, include chemical nature of impurities, stability, and capability of the NM to release ions or other moieties.

### Nanomaterial Characterization

NM characterization generally includes properties such as chemical composition and physical aspects such as particle size, aggregation and agglomeration characteristics, surface chemistry, surface coating, functionalization, and morphology (shape, surface area, surface topology). Many methods are recommended for NM characterization, based on spectroscopic or imaging technologies (Zhu et al., 2013; Lin et al., 2014; Committee et al., 2021). For example, the primary sizes of NM can be determined using transmission electron microscopy. A certain number of NM should be measured, and the size distribution of the particles and aggregates calculated. However, a common set of methods, many of which depend on specialized instrumentation, has not been established. Methods for NM characterization are provided in references (Zhu et al., 2013; Lin et al., 2014; Dusinska et al., 2017; ISO, 2017; Faria et al., 2018; Committee et al., 2021). Ideally two or more different methods are recommended to measure each parameter in order to minimize the risk of artefacts.

The most important characterization is an assessment of NM properties within biologically representative media. This includes providing an understanding of agglomeration under experimental conditions, material stability and evaluation of the transformation of the material during the experiment, e.g., changes to surface chemistry and/or morphology, and material dissolution. Data comparing the physico-chemical characteristics of the NM in the original supplied form and under experimental conditions (i.e., in medium) are likely to be informative for NM effects in actual use situations.

As noted above, agglomeration and aggregation of particles is an important factor that should be addressed and monitored in sample suspensions before and after testing. Toxicological testing is generally valid for un-agglomerated particles, or as expected in real world use, if agglomeration is expected in real use situations. Since agglomeration is more likely at higher doses, agglomeration should be assessed to assist in choosing the higher doses proposed for the test. Due to their high surface energy, NM may also interact with the testing medium or bind to different substances, including proteins in the test medium or in the *in vivo* environment, possibly resulting in altered biological activity. These factors should be considered if relevant to specific routes of exposure, such as effects in the gastrointestinal tract after oral dosing.

Generally, the dynamic light scattering (DLS) technique can be used to characterize the behavior of the NM. Hydrodynamic size and surface charge can be measured using a Zetasizer or another equivalent instrument. Cell uptake of the particles can be confirmed with microscopic images with or without tags such as metals. Chemical and other analyses can be used to identify NM composition.

### Sample Preparation

Describe sample preparation and provide justification for the choice of the suspending medium (vehicle) which should be compatible with the assay used. Due to solubility issues, NM are often present as a dispersion of particles. Information should be provided on handling of the NM, such as sonication of the suspension. Suspensions of the NM test article should be prepared just before use in the assay. Description of NM storage and the potential for change in properties during storage should be considered.

### Dose Selection

Dosing and dose-response assessments are critical factors in the safety assessment of NM. In our review (Elespuru et al., 2018) we noted a lack of a rationale for often excessive amounts/doses of NMs used in genotoxicity assays. Excessive doses may create artifacts that are not representative of real use situations, or even mask a real effect (e.g., reverse dose-response curves, where higher doses have less effect than lower doses). Dose selection is still a difficult issue, without consensus, but dose limits for NM are generally considered lower than those in the OECD guidelines. A rationale for dose choices should be provided using experimental or published data. Exposures expected during actual use of the NM are useful for interpretating results, but alone they are not adequate determinants of dosimetry for safety assessment. Toxicological assessments are customarily conducted at higher than actual use doses in order to compensate for uncertainty, as well as to assure detection of a response that may be missed at lower doses. OECD guidelines indicate dosing limits for specific assays; these exposures should be included in the dose-response if they don’t interfere with the assay or generate artifactual results. Appropriate dose-spacing to inform NM effects is a critical feature of valid testing. When toxicity is observed, doses should range from non-toxic levels to varying toxicity levels up to a maximum recommended in the OECD guideline for the assay being performed, generally based on cytotoxicity in the test system or the onset of agglomeration or aggregation. The assessment of solubility/dissolution rate, dispersion, aggregation, and agglomeration should be considered for each dose. A total dose-range of 20 to 50-fold, with dose spacing chosen based on preliminary experiments, is recommended for the definitive test. Once a dose range is determined in preliminary experiments, a narrowed set of doses varying by approximately 2 to 3-fold should be chosen for the definitive test. OECD guidelines may be informative for dose choices for specific tests, but upper exposure limits for NM may be lower, because of agglomeration and other factors (Wills et al., 2017). Dose limits should be justified by experimental data on dose-related agglomeration, aggregation, inflammatory effects (*in vivo*), or potential artifactual results (impacting the assay or test conditions).

### Uptake/Exposure

A major consideration for a valid *in vitro* NM genotoxicity test is uptake by the cells to indicate cell exposure. Effects of released ions from NM would qualify as appropriate for targeted analysis.

Some NM physicochemical properties may alter transport of chemical agents into cells. For instance, Ag (silver) ions are transported into bacteria, but nano Ag is not taken up (Butler et al., 2015). This paper also demonstrates multiple methods, including the use of flow cytometry in determining uptake of a NM.

Ideally, information on uptake would be provided for the NM and the cell system under study. If uptake studies are possible, they provide valuable information enhancing genotoxicity data, particularly in the case of a negative test. General principles and methods addressing uptake assessment are provided (Hondow et al., 2011; Kettler et al., 2014; Zhang et al., 2015; Behzadi et al., 2017; Wu et al., 2019). If it is not possible to provide experiments demonstrating NM uptake, dose-response experiments should demonstrate toxicity within acceptable parameters of agglomeration, if not to the limits described in the OECD test guidelines. This provides evidence that the material reached the test system and exposure was effective.

For *in vivo* assays, evidence of distribution to target cells or evidence of released ion effects is necessary for a valid test. Validity of a negative result requires evidence that the NM test article reached the target cells. Acceptance of a positive result requires evidence that the NM exposure did not overwhelm the test system, producing artefactual results. For example, abdominal hemorrhage following a large dose may cause systemic toxicity irrelevant to lower doses. Lack of systemic bioavailability in many cases may be adequate evidence of lack of hazard. However, lack of uptake, and possible false negative results, can result from the use of inappropriate test systems (such as those based on the use of bacteria).

### Positive and Negative Controls

Positive controls are designed to demonstrate that the test system is capable of delivering the response or outcome being queried. Although positive NM controls are being sought for several genotoxicity assays, in principle, positive controls do not need to be NM. The most extensive studies of a potential nanoparticle positive control are of WC-Co (Tungsten Carbide Cobalt) by Moche et al. (2015), including studies in gene mutation assays, *in vitro* micronucleus assays and comet assays. Results were significantly positive but somewhat variable. NM genotoxic responses are typically weak. These authors concluded that the mode of action (MOA) was likely via oxidative damage. However, further studies are needed on NM effects. Because positive controls are designed to demonstrate assay integrity, studies with NM test articles are generally performed in assays with standard non-NM positive controls (noted in OECD guidelines for each assay) that produce robust responses in the assays.

Negative controls are the solvent vehicle in which the NM is suspended. Typical negative controls are compatible with the biological test system used, and include water, saline, or cell culture medium. If non-standard vehicles are used, it should be demonstrated in preliminary experiments that the vehicle in use does not affect the test system or outcome of testing.

If a NM is expected of interfering with the assay endpoint measurement or biological response, the positive control could be run with and without the NM to determine an inhibitory effect or interference in the positive control outcome.

### Metabolic Activation

Many carcinogens and genotoxins require metabolic activation to reactive forms that cause diverse genotoxic effects. Thus, for valid safety assessment, genotoxicity testing generally includes sets of tests in the presence and in the absence of a metabolic activating system. Whereas *in vivo* systems contain inherent metabolic activating capability, *in vitro* assays require the addition of an activating system. The standard *in vitro* metabolic activation system consists of a 9,000 × g liver homogenate (S9) from rats treated with phenobarbital/β-naphthoflavone (or other validated inducers), plus cofactors. Chemicals may become more or less reactive/active in the presence of the metabolic activation system. However, few if any NM are known to require metabolic activation for generation of a positive genotoxicity response. In order to save animals, materials and time, we recommend that most NM do not need to be tested with S9 metabolic activation mix, including e.g., metal or polymer NM. However, if metabolic activation is indicated, the standard recipe mix and alternative resources are provided here.

The final concentrations of the co-factors in the S9 mix consists of:

• 5 mM glucose 6-phosphate,
• 4 mM nicotine-adenine dinucleotide phosphate (NADP)
• 8 mM MgCl2
• 33 mM KCl in a 100 mM phosphate buffer at pH 7.4.

S9 fraction and cofactor mixes are available commercially or may be prepared in-house. The freshly thawed (and kept on ice) S9 preparation is mixed with the cofactor pool in defined amounts to result in e.g., 10% S9 and 1X cofactors. This is the S9 mix, which is added to test systems in defined amounts, e.g., into mammalian cell assays at 10% resulting in a final concentration of 1% S9. See Maron and Ames (Maron and Ames, 1983).

## Results: Evaluation and Interpretation of NM Test Results

A test result is considered clearly positive or negative based on the following criteria (Table 1).

**TABLE 1:** Criteria for positive or negative result.

	Clearly positive	Clearly negative
Criteria	1. At least one of the test groups* exhibits a statistically significant increase in the assay endpoint compared to the concurrent negative control	1. None of the test groups* exhibit a statistically significant increase in the assay endpoint compared to the concurrent negative control
	2. Any of the results are outside the distribution of the historical negative control data (e.g., 95% control limits)	2. All results are inside the distribution of the historical negative control data (e.g., 95% control limits)

*Test item, test article or test group: the solution, suspension, or other preparation of the NM added to the test; a test group would be one dose sample among several of the samples in the assay.

“Historical control data” refers to accumulated data from previous experiments.

Both criteria should be met to consider a result clearly positive or negative. There are cases where it is not possible to determine a clearly positive or a negative result. Then, a repeat experiment is recommended with a modified study design to clarify results, for example, with more closely spaced dose levels in the optimum dose range, and/or increased numbers of cultures per dose). Genotoxicity test guidelines typically recommend a dose-response study as a criterion for a clearly positive result. In the case of NM, a dose-response may not be observed. Higher doses where agglomeration occurs may decrease cell uptake and thus lead to an abnormal dose response (Wills et al., 2017). Therefore, a dose-response is not required for a clearly positive result when testing NM. However, dose-response and reproducibility information should be included in the assessment, along with information on NM properties, including e.g., changes in agglomeration as a function of dose. In case a clearly positive or negative result cannot be determined after a repeat of the experiment, results may be considered *equivocal*.

## Discussion

Results should be discussed in terms of technical analysis of the properties of the NM and its characteristics that are relevant to the results. For example, what is the dynamic range of the induced effect, if a positive result is observed? How might the result inform the mode of action, e.g. as a direct or an indirect genotoxic effect? If bioavailability was not achieved in conjunction with a negative result, this should be discussed. What is the impact of this result on hazard consideration of the NM?

## Recommendations Guideline

As noted, we don’t think specific recommendations are appropriate to address NM issues at this time, but the following general recommendations are provided for consideration.

• Scoping: what is the purpose of the testing/assessment?
  ∘ General hazard identification
  ∘ Specific question or focus
• Test selection (from the Genotoxicity test battery adapted to NM, Roadmap (Figure 1)
  ∘ Gene mutation: TK mutation assays: Mouse Lymphoma (MLA), or TK6
  ∘ Clastogenicity (large scale DNA damage): *in vitro* Micronucleus Assay, or MLA
  ∘ *In vivo* assessment: Comet Assay (DNA strand breaks)
• NM assessment
  ∘ Characterization (in the test medium if possible)
     ⁃ Choice of assessments: size, shape, distribution, uniformity, representative photo
     ⁃ Choice of instrumental measurements
  ∘ Sample preparation
     ⁃ Vehicle selection: NM ideally in suspension in bio-compatible vehicle
     ⁃ Potential agglomeration?
     ⁃ Sonication?
  ∘ Dose selection
     ⁃ Dose-range finding study
     ⁃ Dose choice
        • Meets assay requirements (OECD guideline suggested limits may not be applicable)
      • Does not interfere with the assay
      • NM can be separated from the test system after exposure time
  ∘ Exposure assessment
     ⁃ ADME: distribution in animals (if info is available for consideration)
     ⁃ Uptake into cells
     ⁃ Fate of particles
     ⁃ Fate of marker such as ion or element
  ∘ Negative and position controls
  ∘ Data analysis

## Data Availability

Publicly available datasets were analyzed in this study. This data can be found here: Data are referred to in the references.
